# A rich TILLING resource for studying gene function in *Brassica rapa*

**DOI:** 10.1186/1471-2229-10-62

**Published:** 2010-04-09

**Authors:** Pauline Stephenson, David Baker, Thomas Girin, Amandine Perez, Stephen Amoah, Graham J King, Lars Østergaard

**Affiliations:** 1 Department of Crop Genetics, John Innes Centre, Norwich, NR4 7UH, UK; 2 John Innes Genome Laboratory, John Innes Centre, Norwich, NR4 7UH, UK; 3 Plant Science Department, Rothamsted Research, Harpenden, Hertfordshire, AL5 2JQ, UK

## Abstract

**Background:**

The *Brassicaceae *family includes the model plant *Arabidopsis thaliana *as well as a number of agronomically important species such as oilseed crops (in particular *Brassica napus, B. juncea *and *B. rapa*) and vegetables (*eg. B. rapa *and *B. oleracea*).

Separated by only 10-20 million years, *Brassica *species and *Arabidopsis thaliana *are closely related, and it is expected that knowledge obtained relating to *Arabidopsis *growth and development can be translated into Brassicas for crop improvement. Moreover, certain aspects of plant development are sufficiently different between *Brassica *and *Arabidopsis *to warrant studies to be carried out directly in the crop species. However, mutating individual genes in the amphidiploid Brassicas such as *B. napus *and *B. juncea *may, on the other hand, not give rise to expected phenotypes as the genomes of these species can contain up to six orthologues per single-copy *Arabidopsis *gene. In order to elucidate and possibly exploit the function of redundant genes for oilseed rape crop improvement, it may therefore be more efficient to study the effects in one of the diploid *Brassica *species such as *B. rapa*. Moreover, the ongoing sequencing of the *B. rapa *genome makes this species a highly attractive model for *Brassica *research and genetic resource development.

**Results:**

Seeds from the diploid *Brassica *A genome species, *B. rapa *were treated with ethyl methane sulfonate (EMS) to produce a TILLING (Targeting Induced Local Lesions In Genomes) population for reverse genetics studies. We used the *B. rapa *genotype, R-o-18, which has a similar developmental ontogeny to an oilseed rape crop. Hence this resource is expected to be well suited for studying traits with relevance to yield and quality of oilseed rape. DNA was isolated from a total of 9,216 M_2 _plants and pooled to form the basis of the TILLING platform. Analysis of six genes revealed a high level of mutations with a density of about one per 60 kb. This analysis also demonstrated that screening a 1 kb amplicon in just one third of the population (3072 M_2 _plants) will provide an average of 68 mutations and a 97% probability of obtaining a stop-codon mutation resulting in a truncated protein. We furthermore calculated that each plant contains on average ~10,000 mutations and due to the large number of plants, it is predicted that mutations in approximately half of the GC base pairs in the genome exist within this population.

**Conclusions:**

We have developed the first EMS TILLING resource in the diploid *Brassica *species, *B. rapa*. The mutation density in this population is ~1 per 60 kb, which makes it the most densely mutated diploid organism for which a TILLING population has been published. This resource is publicly available through the *RevGen*UK reverse genetics platform http://revgenuk.jic.ac.uk.

## Background

The advent of high-throughput sequencing technologies, vast genomic databases and increasingly powerful genetic tools has had a huge impact on the development of our understanding of the biochemical and developmental networks regulating the multitude of genetic and physiological processes in plants [1]. Insight from studies in the model species, *Arabidopsis thaliana*, is increasingly facilitating our ability to elucidate and beneficially exploit key regulatory processes in relevant crop species. The last decade has seen the development of a number of large-scale 'Reverse Genetics' tools to study the effects of mutations in genes for which the sequence is known. These tools include T-DNA insertion [2], TILLING (Targeting Induced Local Lesions In Genomes) [3] and RNAi technologies [4-6].

TILLING is a reverse genetics tool, which was originally developed for *Arabidopsis *[7] and has subsequently been successfully employed in other plant species as well as animal species (eg. [8-17]). For plants, large mutant populations are generated by the treatment of seed or pollen with a chemical mutagen - most commonly ethyl methane sulfonate (EMS) - that can induce point mutations at a very high density, sufficient to establish a series of allelic mutations in all genes. Amplified sequences are then screened using established high throughput SNP discovery methods.

*Brassica napus *(oilseed rape) is an amphidiploid species containing two diploid genomes originating from a cross between the diploid *Brassica *species, *B. rapa *and *B. oleracea*. Whereas TILLING populations have been described for *B. napus *and *B. oleracea *[13,15], such a resource has not yet been reported for *B. rapa*.

EMS is a mutagenic, teratogenic and possibly carcinogenic organic compound and it is the mutagen of choice for the development of plant TILLING populations [7-15]. It produces random mutations in genetic material by nucleotide substitution; primarily by alkylation on the O^6 ^position of guanine leading to GC→AT transition changes.

Here we describe the development of a TILLING population in *B. rapa *genotype R-o-18 [18]. The effect of EMS on plant growth and fertility in the M_1 _and M_2 _generations is described. Based on the screening of six genes located on different chromosomes, we calculated a mutation density of ~1 per 60 kb and a 97% probability of identifying a stop-codon mutation in a standard screen of 3072 M_2 _plants. This resource therefore comprises an attractive tool for researchers having interests in plant development and especially with regard to phenotypic traits related to improvement of oilseed rape and other crops.

## Results and Discussion

### Choice of model plant

**Figure 1:**
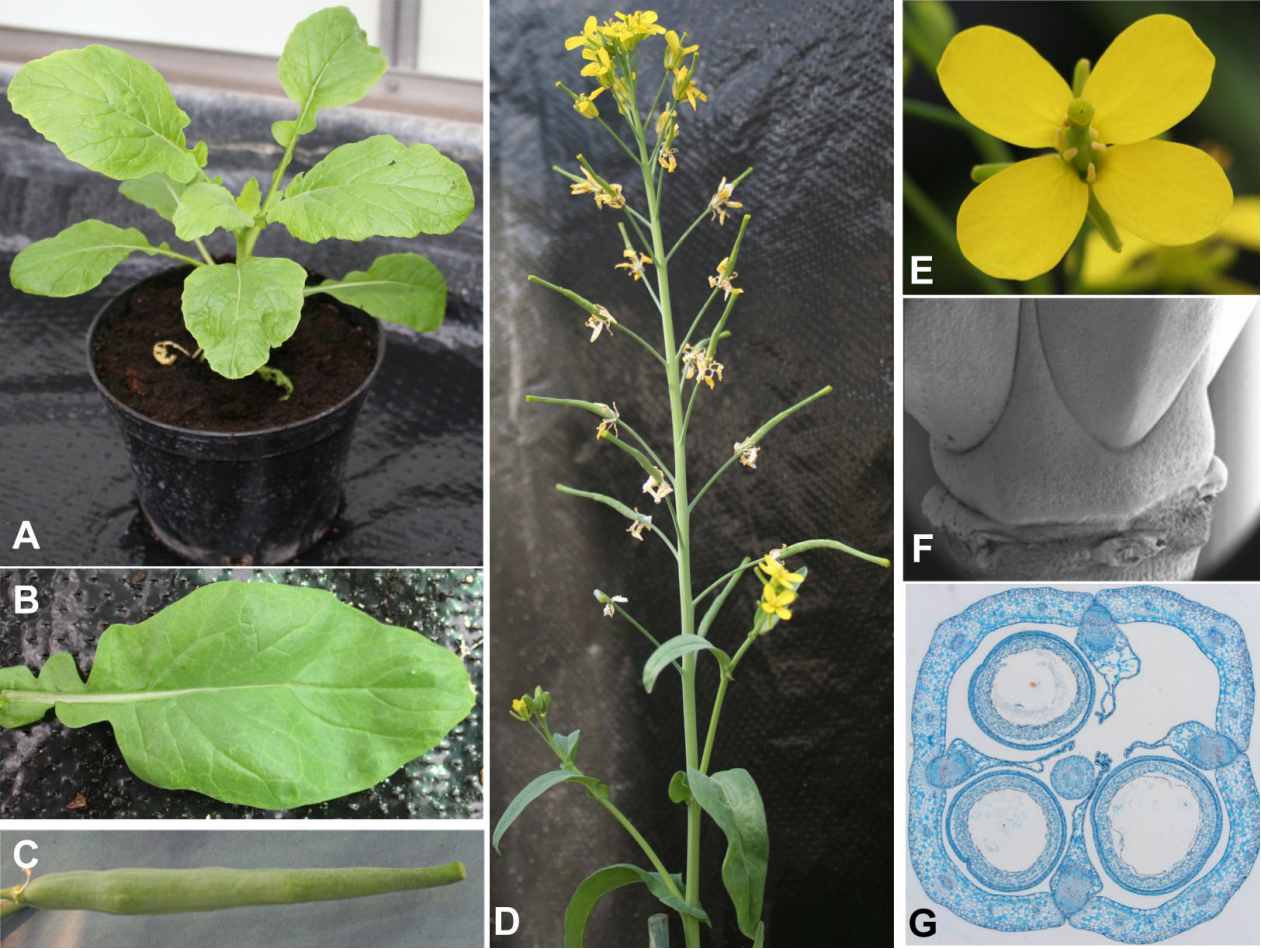
***B. rapa *genotype R-o-18 plant development**. A) Seedling three weeks after sowing, B) Fully expanded rosette leaf, C) mature and fully elongated fruit, D) main shoot of flowering R-o-18 plant seven weeks after sowing, E) open flower, F) scanning electron micrograph of the base of gynoecium at anthesis, G) cross section of fully elongated R-o-18 fruit.

R-o-18 (Figure 1) is an inbred line of the *Brassica rapa *subsp. *trilocularis *(Yellow Sarson) with transparent seed coat [18] closely related to *B. rapa *oilseed crops grown in Pakistan [19]. It was favoured as the genetic system for the TILLING population described here due to the following features: 1) it is diploid, which simplifies the genetics and reduces the potential for genetic redundancy, 2) *B. rapa *genome sequencing is ongoing and sequence information on the target gene is a prerequisite for reverse genetics, 3) R-o-18 is self fertile and produces a large number of seeds per plant (this is in contrast to several other *B. rapa *varieties such as the subspecies *chinensis *and *pekinensis*) and 4) the similarity in growth pattern to both the model plant *Arabidopsis thaliana *and oilseed rape varieties makes it an attractive system for fundamental research as well as studies related to yield and quality traits in *B. napus*. Such traits include seedling establishment, plant architecture, seed oil composition, fruit development, nutrient and water use efficiency and disease resistance.

### Optimising mutagen dosage

**Figure 2:**
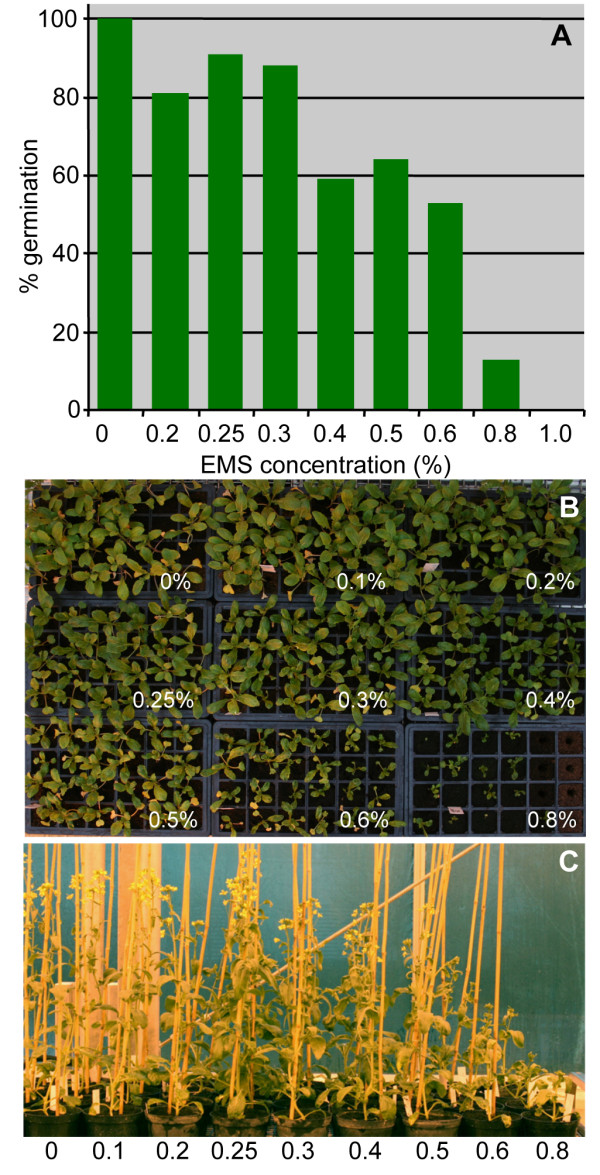
**Effect of EMS treatment on M_1 _plant growth**. Titration of A) germination frequency, B) seedling establishment and C) plant vigour in response to increasing EMS concentrations (x-axis).

Under optimal mutagenesis conditions, individuals of an EMS mutant population carry a high mutation load but remain vigorous and fertile. It is important, therefore, to determine the level of mutagen treatment necessary to achieve the maximal mutation load. We carried out incubations of R-o-18 seeds with EMS concentrations ranging from 0 to 1% (Figure 2).

EMS is a highly volatile and unstable compound and we have previously observed variation in the activity between batches. In order to avoid having to repeat the optimisation experiment with a different EMS batch after the titration, we used the same fresh batch of EMS to treat 5,000 seeds for each concentration in the 0.2-0.5% range (expected to be most relevant for producing the population based on previous experience) and 200 seeds at the concentrations outside this range.

After incubation and washes, the seeds were sown in soil and kept at 7°C for six days. Following a further six days in the glass house, germination frequency was established. Germination was hardly affected by treatments up to 0.3%. However, at 0.4% a marked decrease was observed and at 1% EMS none of the seeds germinated, indicating that the EMS treatment had been effective.

For *Medicago truncatula *mutagenesis, it was previously reported that an EMS concentration at the point where germination begins to become compromised is optimal for obtaining a large mutation load while maintaining vigorous and fertile plants [20]. For the *B. rapa *population we therefore decided to use seedlings derived from the 0.3% and 0.4% EMS treatments for population development, and the resulting two populations will subsequently be referred to as the '0.3% population' and the '0.4% population'.

Although, only seedlings from the 0.3% and 0.4% EMS-treated seeds were used to make up the M_1 _generation of the mutant population, a subset of the germinated seedlings from the remaining concentrations in the titration experiment were allowed to grow on. As shown in Figure 2b and 2c, the higher concentrations of EMS also inhibited seedling establishment as well as plant growth.

**Figure 3:**
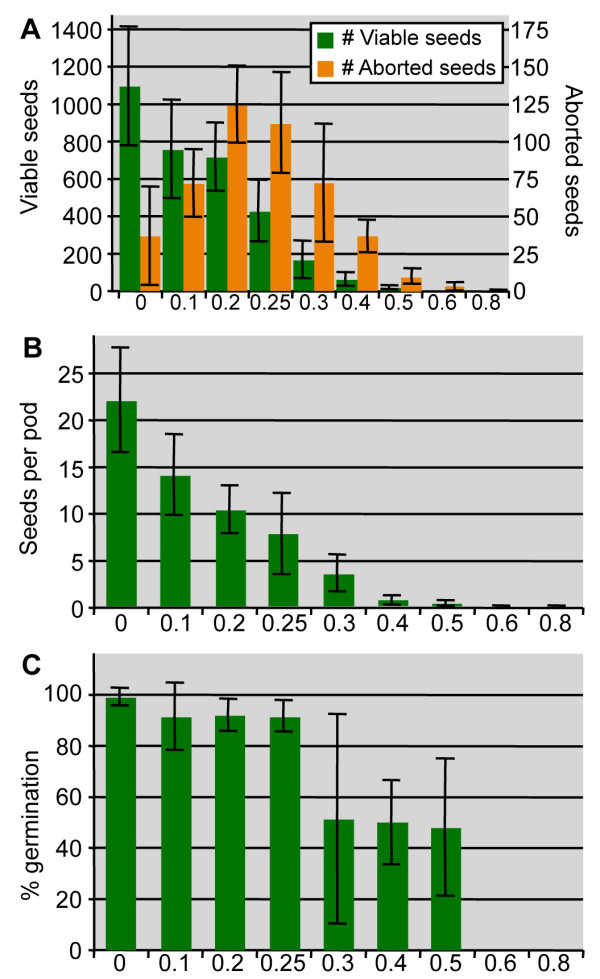
**Effect of EMS treatment on fertility**. The effect of increasing EMS concentrations on A) viable and aborted M_2 _seeds, B) number of M_2 _seeds per pod, and C) germination of M_2 _seeds. Bars represent standard deviations of mean.

Twenty M_1 _plants from each concentration were grown to maturity to establish the overall population fertility. The total number of viable seeds decreased gradually throughout the concentration range; the number of aborted seeds first increased as expected and then decreased at the higher concentrations probably due to effects on early ovule development (Figure 3a). The number of seeds per pod also decreased gradually with a severe reduction already apparent at 0.3% and 0.4% EMS (Figure 3b). The viability of the isolated seeds was established in a germination assay (Figure 3c). Despite a clear reduction in seed number at the low EMS concentration, no effect was observed on M_2 _seed viability until the 0.3% EMS level. This could be due to the lack of homozygosity at a sufficient number of loci. However, the huge variation in seed viability observed at the 0.3% EMS level, suggests that a high mutation frequency has been reached at this concentration. In total, we obtained M_2 _seeds from 3,464 and 1,564 M_1 _plants from the 0.3% and 0.4% populations, respectively.

### M_2 _population and TILLING platform

When treating the seeds with the mutagen, a subset of cells in the shoot apical meristem of the embryo will carry the mutations on to the next generation. This provides an opportunity for multiple cell lineages to be subject to a different spectrum of mutations, and it is therefore possible for gametes arising from different floral primordia to carry a distinct subset of mutant alleles [21].

Since *B. rapa *plants are larger, generate less seed and have a longer life cycle relative to, for example, *Arabidopsis thaliana *ecotypes Col-0 and L-*er *(5-6 months versus 6 weeks from seed to seed), it is desirable to minimise the number of plants necessary to build a useful resource. Under optimal conditions, we estimated that a sufficient number of the mutations would be recovered by using material from two M_2 _plants from each of the ~5,000 M_1 _plants assuming a similar number of progenitor cells as in *Arabidopsis*. Ten seeds from each M_1 _individual were planted to increase the probability that at least two would germinate.

During growth of the M_2 _population a number of phenotypes were observed; the percentage of M_2 _families with albinos was 4.5% for both the 0.3% and 0.4% populations and we observed a plethora of morphological defects at developmental stages, ranging from seedlings to the fruit stage. Galleries of selected phenotypes are shown in Additional files 1, 2, 3 and 4.

### TILLING platform design

For most M_2 _families, 5-10 seeds germinated and in these cases we always took leaf tissue from the two most healthy-looking individuals and discarded the rest. In this way, we obtained vigorous and mostly fertile plants, while expecting to maintain the high mutation level in a heterozygous state. The plants were subsequently bagged to prevent pollination between plants and M_3 _seeds were harvested.

Upon harvesting, we recorded the fertility and found that 9.6% of the M_2 _plants from the 0.3% population and 27.9% of the M_2 _plants from the 0.4% population failed to set seeds suggesting a higher mutation load in the 0.4% population.

DNA was isolated from the tissue, the concentration of DNA accurately determined and stocks normalised to ensure that DNA pools were balanced such that all individual lines were equally represented within the pools.

We used a standard one-dimensional pooling strategy where each M_2 _line is represented only once in a single pool, with each pool comprising DNA from eight M_2 _lines. Such a design is ideally suited to high throughput mutation detection. Specifically, eight-pools of 6,912 M_2 _plants originating from the 0.3% population were distributed in nine 96-well plates (DNA from 768 M_2 _plants per plate), whereas DNA pools from 2,304 M_2 _plants from the 0.4% population were distributed in three plates.

Mutations in genes of interest are detected by *Cel*1 digestion at a mismatched base pair [22,7]. For the *B. rapa *population described here, identification of digested fragments were carried out on an ABI3730 sequencer using fragment lengths of ~1 kb and a previously established protocol [14]. Individual M_2 _lines from pools identified as containing a mutant allele were subsequently sequenced in order to confirm the presence of the mutation, reveal its identity and to identify the M_2 _line carrying the mutation. Since the *Cel*1-digested product is verified with labelled primers from both ends [14], the level of false positives is essentially zero.

### EMS mutagenesis and the genetic code

When initiating a TILLING screen in a particular gene of interest, it is useful to analyse the coding region with reference to the genetic code. Only a subset of all combinations of amino acid changes are achievable when using EMS as a mutagen. Firstly, eight out of the 64 codons (12.5%) are unaffected by EMS-induced mutations because they do not contain guanine or cytosine. Secondly, out of the 96 positions that can be mutated (G→A or C→T) within the genetic code, 33 would not lead to an amino acid sequence change (silent mutations). Of the remaining 63 mutable positions of the genetic code, 58 would give rise to 26 amino acid substitutions (mis-sense mutations) and 5 would result in stop codons (nonsense mutations). Nine of the possible amino acid changes (corresponding to mutations at 21 out of 58 sites) result in chemically similar amino acids being incorporated which in many cases would be less likely to alter the function of the encoded protein significantly.

Mutations leading to premature stop codons are often desirable as they are expected to provide a dramatic reduction in gene function, especially when proximal to the 5' end of the open reading frame. However, out of the 96 mutable positions, there are only five ways in which a stop codon can be obtained. These comprise the two glutamine codons (CAA and CAG), one of the six arginine codons (the C of CGA) and the tryptophan codon (TGG) for which G→A mutations at either position will generate a stop codon. The genetic code therefore has considerable robustness built-in, which minimises the potential biological effect of point mutations. It may therefore be beneficial to target the analysis of the gene of interest to a region in the sequence where it is possible to realise fully the potential of mutations that may reduce or abolish the activity of the encoded protein. To assist us in this analysis, we use the software package CODDLE [3], which is a programme designed to identify areas within the gene with highest probability of affecting gene function when mutated by EMS.

### Amplicon selection

Selecting a suitable amplicon for mutation detection is a pre-requisite for establishing efficient and successful screens, and several considerations need to be taken into account: 1) In the large majority of cases, it will be advantageous to include as much coding sequence as possible and avoid intron or intragenic sequence. 2) Repetitive sequence may cause '*Taq *slippage' which could delete or insert extra repeats. This will lead to artifactual mismatches between wild type and 'slippage' strands, which may become substrates for the *Cel*1 nuclease. 3) As mentioned above, only a subset of codon-changes is likely to have dramatic effects on gene activity. It is therefore advisable to identify the region with most potential for generating stop codons and significant amino acid changes. 4) Finally, it is important to test for paralogue-specificity as up to three copies of each single-copy *Arabidopsis *gene may be present in the diploid *Brassica *genome [23,24]. When designing primers for the region of interest, it is therefore essential to verify that this primer set only amplifies the expected sequence before initiating the TILLING screen.

### TILLING assays

We identified six genes that are located on different *B. rapa *chromosomes. These were expected to be orthologues of the *Arabidopsis REPLUMLESS *(*RPL*; At5 g02030) [25], *INDEHISCENT *(*IND*; At4 g00120) [26] and *METHYLTRANSFERASE1 *(*MET1*; At5 g49160) [27] genes and were named *BraA.RPL.a*, *BraA.RPL.b*, *BraA.RPL.c*, *BraA.IND.a*, *BraA.MET1.a *and *BraA.MET1.b*, respectively, according to the accepted gene nomenclature system for the *Brassica *genus [28]. These *B. rapa *gene sequences were isolated using the *Arabidopsis thaliana *Integrated Database http://atidb.org as described in the Methods section.

We performed TILLING on ~1 kb amplicons from the six genes on the 0.3% population and for two genes on the 0.4% population. Based on the number of mutations identified for the individual genes, the TILLING populations were characterised with respect to mutation density and load (Table 1). The density was calculated at ~1 per 56 kb for the 0.3% population and ~1 per 67 kb for the 0.4% population after normalisation to the average 35% GC level for the *B. rapa *genome [29].

**Table 1:** Results from eight TILLING assays in the *B. rapa *mutant population.

Gene name	EMS (%)	Length (bp)	GC (%)	Mutations detected	Screened M_2 _plants	Est. mutations per M_2 _plant*	M_2 _plants in population	Mutation density**	Expected mutations kb^-1...^
*BraA.RPL.a*	0.3	1007	47	21	768	13577 (10111)	6912	1/37 (1/49)	188 (164)
*BraA.RPL.b*	0.3	1072	45	149	4608	15082 (11730)	6912	1/33 (1/43)	209 (190)
*BraA.RPL.c*	0.3	1001	47	132	4608	14309 (10656)	6912	1/35 (1/47)	198 (173)
*BraA.IND.a*	0.3	1004	41	35	3072	5674 (4844)	6912	1/88 (1/103)	78 (78)
*BraA.MET1.a*	0.3	1104	48	94	3072	13858 (10105)	6912	1/36 (1/49)	191 (164)
*BraA.MET2.b*	0.3	1007	47	89	3072	14385 (10712)	6912	1/35 (1/47)	199 (173)

**Relevant averages**						**12814 (9693)**		**1/44 (1/56)**	**177 (157)**

*BraA.RPL.b*	0.4	1072	45	57	2304	11539 (8975)	2304	1/43 (1/56)	53 (48)
*BraA.RPL.c*	0.4	1001	47	40	2304	8672 (6458)	2304	1/358(1/77)	40 (35)

**Relevant averages**						**10106 (7717)**		**1/51 (1/67)**	**47 (42)**

* The estimated number of mutations per plant was calculated using an estimated genome size of 500 Mb for *B. rapa *[30]. Numbers in brackets were obtained after normalising to the average 35% GC content for the *B. rapa *genome [29].

** Mutation density based on estimated number of mutations per plant divided by the 500,000 kb

genome. Numbers in brackets were obtained after normalising to the average 35%

GC content for the *B. rapa *genome [29].

*** The number of expected mutations per kb in the population was obtained by extrapolating the

number of detected mutations in a subset of the M_2 _plants to the whole population.

Numbers in brackets were obtained after normalising to the average 41% GC content for *B. rapa*

exons [29].

This density is the highest reported for a TILLING population in any plant or animal diploid species. Only in populations of tetraploid and hexaploid wheat [10,11] and the amphidiploid *B. napus *[13] have higher mutation densities been obtained.

Based on the stronger effect of the 0.4% EMS treatment on fertility, seed viability and general plant development compared to 0.3% EMS (Figure 2 and 3), it was unexpected that the number of mutations in the 0.4% population was not higher (Table 1). One explanation for this may be the increased number of homozygous loci observed in M_2 _plants of the 0.4% population (Table 2) and hence higher potential for lethality. We speculate that this difference in heterozygotes: homozygote ratio may be due to the characteristic development in R-o-18 plants of two to four individual racemes derived from different progenitor cells. As a consequence, flowers on different racemes may have a different set of mutations, and since the whole plant is bagged, outcrossing between flowers of different progenitor origin is free to occur. With a decrease in fertility between the 0.3% and 0.4% populations, a higher number of 0.4% plants will have sterile racemes, thereby leading to higher incidents of self-pollination events. We believe this is reflected in the higher occurrence of homozygous plants in this population (Table 2).

**Table 2:** Distribution of mutation classes.

		Mutation class			
			
Gene name	(%) EMS	silent	mis-sense	nonsense	het/hom ratio
*BraA.RPL.a*	0.3	8	12	1	9.5
*BraA.RPL.b*	0.3	30	46	0	8.5
*BraA.RPL.c*	0.3	27	71	0	7.2
*BraA.IND.a*	0.3	10	22	1	15.5
*BraA.MET1.a*	0.3	11	4	2	16.0
*BraA.MET2.b*	0.3	4	12	1	16.0

**Total**		**90**	**167**	**5**	**12.1**

*BraA.RPL.b*	0.4	2	15	2	5.3
*BraA.RPL.c*	0.4	13	12	0	5.3

**Total**		**15**	**27**	**2**	**5.3**

Numbers of silent mis-sense and nonsense (stop codon) mutations are shown for the eight TILLING assays. The het/hom ratio refers to the number of mutations that were detected as heterozygous divided by the number of homozygous mutations.

Using 500 Mbp as an approximate genome size for *B. rapa *[30], it was deduced by extrapolation that each plant contains close to 10,000 mutations (Table 1). One might expect this level of mutations to be lethal. However, as only 11% of the genome is coding sequence [29], we expect no more than 1,100 point mutations within exons of which about 700 will have the potential to cause amino acid changes and approximately 50 could introduce new stop codons. With the high heterozygotes: homozygote ratio (Table 2) and a high level of redundancy, it may therefore not be totally unexpected that it is possible to generate a large number of M_2 _plants with such a high mutation density.

**Figure 4:**
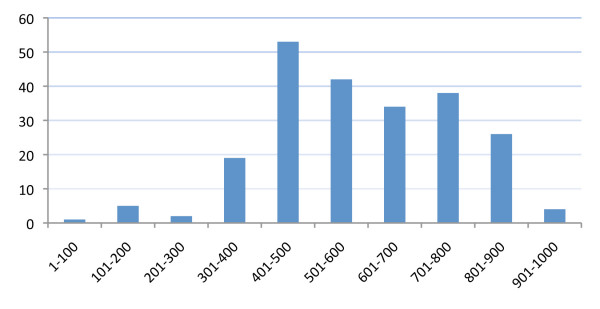
**Distribution of mutations in the amplicons as a function of scaled fragment coordinates**. The length of the TILLed fragments from *BraA.RPL *and *BraA.IND.a *genes were scaled to 1000 bp and the positions of each mutation calculated on this scale. The detected mutations are not equally distributed within the amplicons. Mutations are scarce close to the primers and appear more frequently towards the centre.

The true mutation density is likely to be even higher than calculated here as only five of the total of 617 detected mutations were found within 100 bp from the fragment ends (Figure 4). The difficulty in detecting mutations close to the TILLING primers has been reported previously when LI-COR sequencers are used and 80 bp are now routinely omitted from both ends when calculating mutation densities using this technique [31]. Although a similar effect is observed with the ABI3730 sequencer used here, the mutation-density calculations are based on the actual numbers obtained in the assays and therefore represent an estimation of the minimal density.

Mutations in the *BraA.RPL *and the *BraA.IND.a *genes were asymmetrically distributed along the amplicons (Figure 4). This likely reflects the location of the 5' primers in the *BraA.RPL *genes approximately 300 bp upstream of the start codon in an AT-rich non-coding region, necessary to ensure locus specificity. At the 3' end, however, the primer was positioned within the first exon in an area of more mutable sites (higher GC content).

The high number of plants in this population further contributes to its potential value as an efficient reverse genetics resource. In a standard assay, 3,072 M_2 _plants are screened which corresponds to a third of the combined populations. From our experiments, an average of 68.2 mutations will be recovered when analysing a 1 kb fragment (41% GC on average in *B. rapa *exons [29]) with a 97% probability of obtaining a stop-codon mutation according to the formula:

However, even if the desired mutations are not obtained in the first screen, there is still a further two thirds of the total number of lines available to be tested. In fact, extrapolation of the numbers obtained in the TILLING assays reported here shows that mutations in approximately half of the Gs or Cs in the *B. rapa *genome may exist in this population:

Bearing these numbers in mind, it is therefore surprising that no nonsense mutations were detected within the first 3,072 M_2 _plants for two out of the six amplicons tested here (*BraA.RPL.b *and *BraA.RPL.c *in Table 1). Screening an additional 1,536 M_2 _plants also did not result in any stop codon 'hits', whereas two nonsense mutations were detected for *BraA.RPL.b *after screening the 2,304 plants from the 0.4% population (Table 2).

It is unlikely that this discrepancy is due to lethality of nonsense mutations in these genes. Firstly, we have identified three closely related paralogues suggesting that these genes may function redundantly. Secondly, mis-sense mutations in each individual do not have any detectable effect on plant development (data not shown).

These observations suggest that the lack of nonsense mutation detection in these amplicons is due to limitations of the detection method, which may be related to features in the DNA sequence.

### Gene Redundancy

Although *B. rapa *is diploid, it is still a paleopolyploid having undergone an ancient triplication event [23,24,32,33]. Therefore one can expect to find up to three paralogous genes of each single-copy *Arabidopsis *gene in the *B. rapa *genome. Subfunctionalisation may have evolved in some cases. However, a high level of functional redundancy among the paralogues probably exists, and it may therefore be necessary to combine mutants to observe the desired effect. It is likely that this redundancy between paralogues allows *B. rapa *to harbour such a high mutation density compared to other diploid species. Another factor may be the high level of heterozygosity obtained especially in the 0.3% population, which to our knowledge is higher than for any other TILLING populations reported. The strategy of selecting healthy-looking M_2 _plants for the population will have contributed to this. One might predict that a significant number of M_3 _plants would be severely impaired in development due to a high level of homozygosity. In these cases it is recommended to carry out a backcross to wild type in the M_2 _generation to remove part of the background.

In accordance with the redundancy argument, we did not observe any developmental defects resulting from mutations in individual *BraA.RPL *paralogues. In contrast mis-sense and nonsense mutations in the *BraA.IND.a *gene, which is a single-copy gene in *B. rapa*, result in indehiscent phenotypes as in *Arabidopsis *(T. Girin, P. Stephenson, C. M. P. Goldsack, S. Perez, N. Pires, P. A. Sparrow, T. A. Wood and L. Østergaard - manuscript accepted). *B. rapa *therefore appears to provide a highly suitable compromise between being able to accommodate a high mutation density, whilst still presenting visible phenotypes (see also Additional Files 1, 2, 3 and 4). This is in contrast to reports in *eg*. hexaploid wheat where a high mutation density is achieved, but visible phenotypes are rare [10].

### Linking mutation to phenotype

A classical backcrossing programme to remove the undesirable background mutation load is a prolonged procedure, which is expensive in both time and resources. This is especially true where a genus such as *Brassica *produces large plants with a relatively long generation time. Each backcross generation reduces the mutation load by 50%; so reducing the number of mutations from 10,000 to ten will take ten generations of backcrossing and genotyping (10,000 × 0.5^10 ^= 10).

As an alternative to embarking on such a programme, we instead assess the correlation between mutation and phenotype by comparing homozygous recessive mutants to heterozygotes and homozygous wild-type sibling plants in a segregating population for which the background mutations are the same. A 100% correlation between homozygous mutants and phenotype would lend strong support to the hypothesis that the mutation in the 'TILLed' gene is responsible for the phenotype. Moreover, we also aim to obtain allelic series of independent mutations in the same gene, where related phenotypic variants would strongly associate the phenotype with the gene, thereby avoiding the necessity for a lengthy backcrossing scheme. Detailed description on both of these approaches is provided in [34].

## Conclusions

Here we describe the development of a TILLING population in the *Brassica rapa *genotype R-o-18 suitable for reverse genetics studies. The high mutation density in this diploid species makes it an attractive genetic system for studying plant development and especially for obtaining mutations contributing to phenotypic traits related to crop improvement of oilseed rape. With imminent availability of a complete *B. rapa *genome sequence expected in the very near future, this resource will have particular appeal since time-consuming gene isolation and design of paralogue-specific primers will become a relatively straightforward informatic exercise.

This population is publicly accessible and available via the *RevGen*UK reverse genetics platform http://revgenuk.jic.ac.uk.

## Methods

### Mutagenesis and plant growth conditions

Seeds of the *Brassica rapa *genotype, R-o-18 (age 6-12 months since harvesting) were used in this work. For the negative control and 0.1%, 0.6%, 0.8% and 1% concentrations 200 seeds were treated in 10 ml solution in 50 ml Falcon tubes. For the 0.2%, 0.25%, 0.3%, 0.4% and 0.5% concentrations 5,000 seeds were treated in 250 ml (divided into 25 50 ml Falcon tubes for each of these concentrations). Seeds were soaked in 0.02% Tween 20 for 30 minutes prior to the addition of the EMS and incubated overnight (16 hours). 50 ml Falcon tubes with 200 seeds in each were turned end over end causing the seeds to tumble through the solution ensuring that all the seeds were exposed equally to the EMS without incurring too much physical damage. After treatment the seeds were washed ten times with 0.02% Tween 20 and then mixed with fine grade vermiculite to facilitate their even distribution onto 348 mm × 220 mm seed trays. Seeds were sown at an approximate density of 200 seeds/tray and kept at 7°C with no light for six days before being transferred into the glasshouse at 18°C with 16 hours light. After a further six days the percentage of germination was recorded. These M_1 _seedlings were transplanted first into 24-well modules and then into 1L pots in John Innes no. 2 compost. The developing plants were bagged individually before they flowered using perforated bread bags (Packaging Company Unit, UK) to prevent cross-pollination, and allowed to proceed to maturity.

Dried pods were threshed and the M_2 _seeds from each line were deposited in the John Innes Centre seed-store (1.5°C, 7-10% relative humidity) to ensure their long-term viability. Ten seeds from each line were sown into 1L pots and transferred directly to the glasshouse. After germination the number of albino plants was recorded along with a range of other notable seedling and early leaf variant phenotypes. Each line was then thinned out to two 'healthy' plants per M_2 _line, leaf samples were taken for DNA isolation, and the plants were bagged individually and allowed to grow to maturity. Plants were harvested, threshed and M_3 _seed was placed in the seed-store.

### DNA isolation, normalisation and pooling strategy

Leaf material was collected from each M_2 _plant selected for progression through to M_3 _seed and transferred directly into Qiagen racks on dry ice. DNA isolation was carried out using the DNeasy Plant 96 Qiagen Kit for 96 samples following the manufacturer's instructions (Qiagen, UK). DNA concentrations were determined using PicoGreen (Molecular Probes, Invitrogen Corporation, Carlsbad, California, USA) against a universal DNA concentration standard on a Tecan Genios plate reader. All samples were normalised to 0.5 ng/μl (diluted in deionised water).

A simple one-dimensional eight-fold pooling strategy was employed using a Xiril liquid handling robot (Xiril AG, Hombrechtikon, Switzerland). Final DNA concentration in an eight-pool was 0.5 ng/μl, and 5 μl of eight-pool DNA were used in a TILLING reaction. This leads to a total of 2.5 ng DNA (~0.3 ng from each M_2 _line) in the TILLING reaction.

### Primer design

Primers were designed using the CODDLE (codons optimized to discover deleterious lesions; http://www.proweb.org/coddle) programme [3], combined with the PRIMER3 tool [35] to define the best amplicon for TILLING, aiming for a predicted primer T_m _of 60-70°C. The primers used in this work are listed in Additional File 5. CODDLE identifies areas within the gene which have the highest probability of affecting gene function when mutated by EMS, and scores possible mis-sense and nonsense changes.

### TILLING

Mutant detection was carried out by *Cel*1 digestion followed by analysis on a capillary ABI3730 sequencer (Applied Biosystems, Foster City, California, USA) as described in [14].

### Gene isolation and sequencing

The *Arabidopsis thaliana *Integrated Database (AtIDB - http://atidb.org) was used to blast the *Arabidopsis *genes, *REPLUMLESS *[25], *INDEHISCENT *[26] and *METHYLTRANSFERASE1 *[27] against a database of *B. rapa *BAC end sequences that have been mapped onto the *Arabidopsis *genome. BAC clones likely to contain the orthologous gene were identified based on synteny and primers were designed to sequence the genes directly on purified BAC DNA. No BACs were identified harbouring the *BraA.MET1 *genes. Instead these sequences were obtained from R-o-18 on a sequence homology-approach based on a previous publication [36].

## Authors' contributions

PS performed the EMS treatments and analysed the effect of titration. PS planned, organised and performed the planting, bagging, harvest and seed collection of the M_1 _and M_2 _generations and of tissue isolation for DNA extraction from M_2 _plants. DB set up the TILLING platform and performed the screens. TG isolated and sequenced the *BraA.RPL *genes, AP the *BraA.IND *gene and SA and GJK the *BraA.MET1 *genes. PS and LØ analysed the data and wrote the manuscript. All authors read and approved the final manuscript.

## Supplementary Material

Additional file 1 **Defects in vegetative development**. Examples of phenotypic defects during vegetative development observed in M_2 _generation. Click here for file

Additional file 2 **Defects in leaf development**. Examples of phenotypic defects during leaf development observed in M_2 _generation. Click here for file

Additional file 3 **Defects in inflorescence development**. Examples of phenotypic defects during inflorescence development observed in M_2 _generation. Click here for file

Additional file 4 **Defects in fruit development**. Examples of phenotypic defects during fruit development observed in M_2 _generation. Scale bar: 1 cm Click here for file

Additional file 5 **Oligonucleotides**. List of oligonucleotides used in the TILLING assays. Click here for file
