# Supplementary figures and images for: A rich TILLING resource for studying gene function in Brassica rapa
Source: BMC Plant Biol. 2010 Apr 9;10:62. doi: 10.1186/1471-2229-10-62 (PMC2923536; doi:10.1186/1471-2229-10-62)

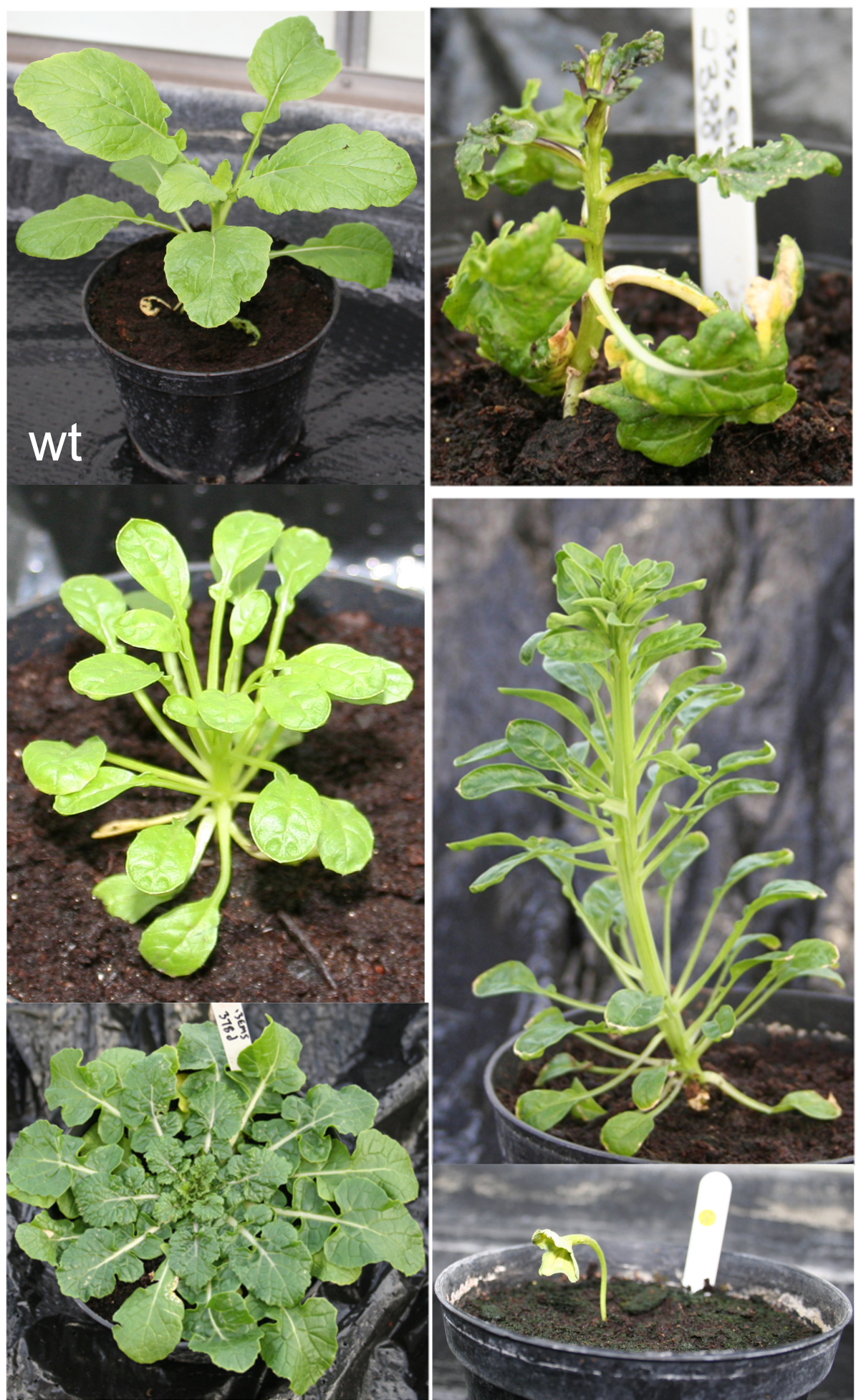

Supplement: Additional file 1 — Defects in vegetative development. Examples of phenotypic defects during vegetative development observed in M2 generation. [file 1471-2229-10-62-S1.JPEG]

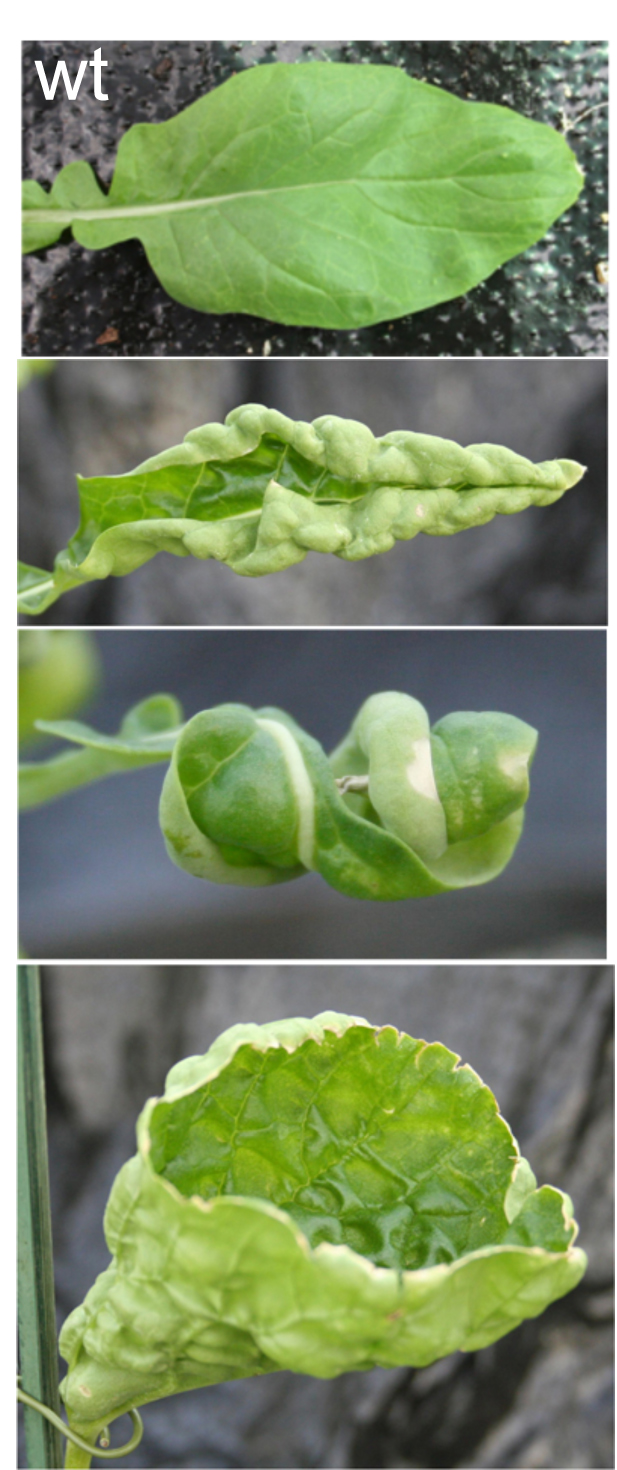

Supplement: Additional file 2 — Defects in leaf development. Examples of phenotypic defects during leaf development observed in M2 generation. [file 1471-2229-10-62-S2.JPEG]

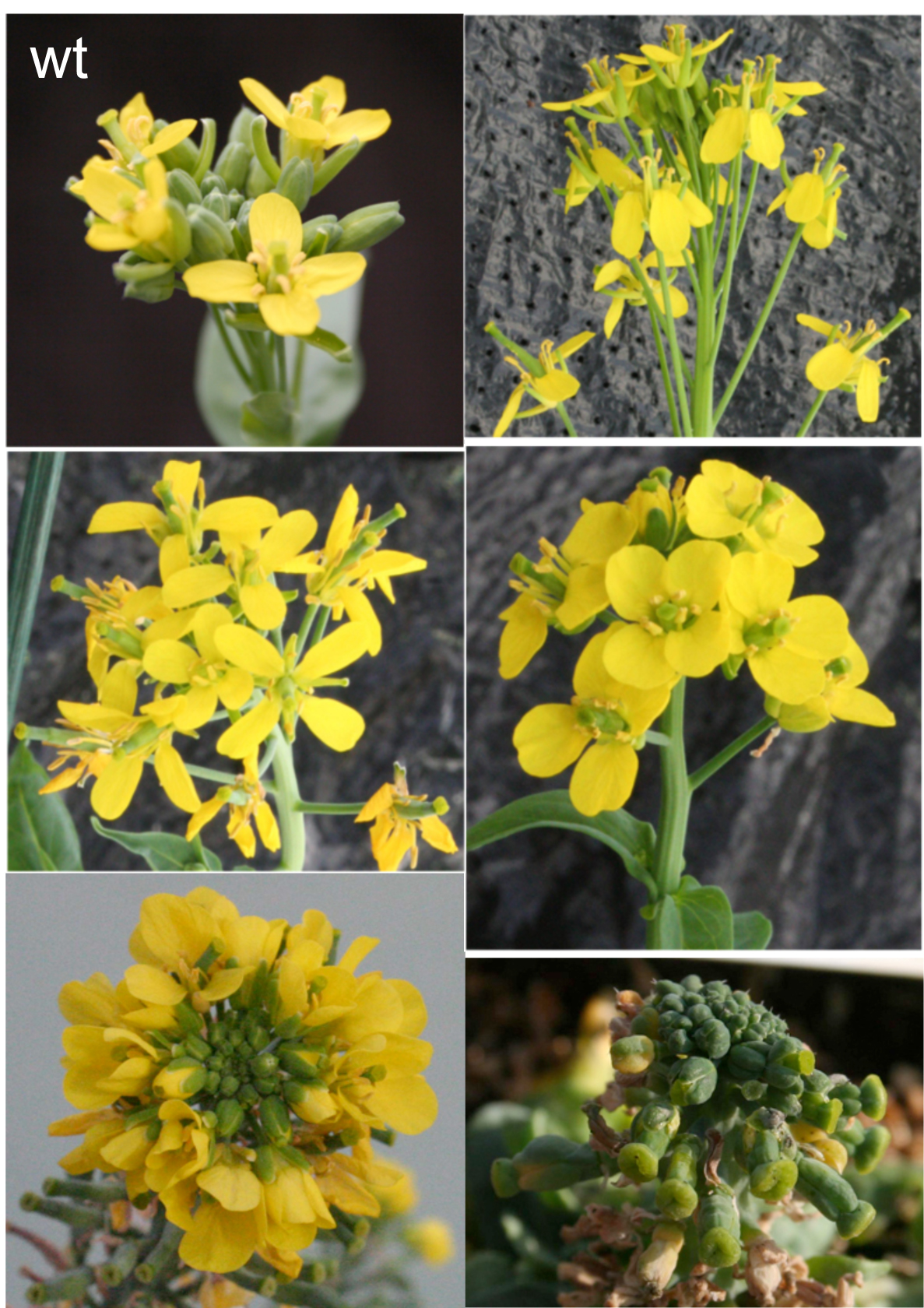

Supplement: Additional file 3 — Defects in inflorescence development. Examples of phenotypic defects during inflorescence development observed in M2 generation. [file 1471-2229-10-62-S3.JPEG]

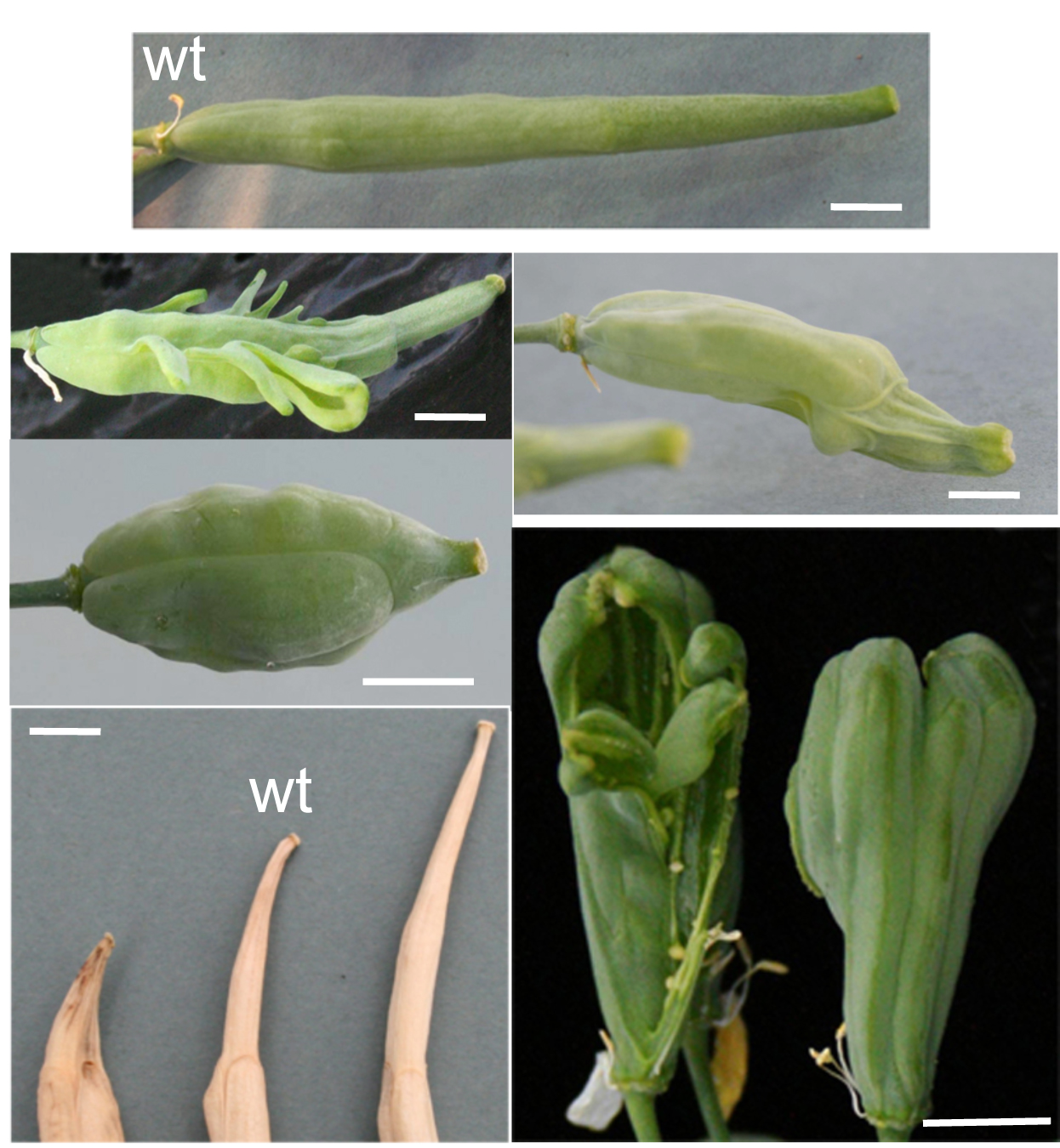

Supplement: Additional file 4 — Defects in fruit development. Examples of phenotypic defects during fruit development observed in M2 generation. Scale bar: 1 cm [file 1471-2229-10-62-S4.JPEG]
