# Supplementary material for: A rich TILLING resource for studying gene function in Brassica rapa
Source: BMC Plant Biol. 2010 Apr 9;10:62. doi: 10.1186/1471-2229-10-62 (PMC2923536; doi:10.1186/1471-2229-10-62)
Supplement: Additional file 5 — Oligonucleotides. List of oligonucleotides used in the TILLING assays. [file 1471-2229-10-62-S5.PDF]

Gene: *BraA.RPL.a*:

BraA_RPL_a_F: 5'-CGGACGGAATCACGTAGATAA-3'

BraA_RPL_a_R: 5'-TCATCGAGCATGGAGATGAG-3'

Gene: *BraA.RPL.b*:

BraA_RPL_b_F: 5'-CCGCACGTAAAACTTCTTCA-3'

BraA_RPL_b_R: 5'-CTTGCCATTTTCTCCTACGC-3'

Gene: *BraA.RPL.c*:

BraA_RPL_c_F: 5'-TGGATCACATACGAGAGAGCA-3'

BraA_RPL_c_R: 5'-TCCAGCATGGAGATGAGCTT-3'

Gene: *BraA.IND.a*:

BraA_INDa_F: 5'-CACGCACTACATTGACATGA-3'

BraA_INDa_R: 5'-TCGGAAAACTTGCATAAGAT-3'

Gene: *BraA.MET1.a*:

BraA_MET1A_F: 5'-CCCTTGCGCTCTAATTGTTC-3'

BraA_MET1A_R: 5'-TTAGGCCGCTGCATAGTTTT-3'

Gene: *BraA.MET1.b*:

BraA_MET1B_F: 5'-ATGATACCGACGGAGTTTCG-3'

BraA_MET1B_R: 5'-ACGGCATAGAGGTAGCAGGA-3'
